# Tabu Search and Machine-Learning Classification of Benign and Malignant Proliferative Breast Lesions

**DOI:** 10.1155/2020/4671349

**Published:** 2020-02-27

**Authors:** Habib Dhahri, Ines Rahmany, Awais Mahmood, Eslam Al Maghayreh, Wail Elkilani

**Affiliations:** ^1^College of Applied Computer Sciences (CACS), Al-Muzahimiyah Branch, King Saud University, Riyadh, Saudi Arabia; ^2^Faculty of Sciences and Technology of Sidi Bouzid, University of Kairouan, Kairouan, Tunisia; ^3^Computer Science Department, Yarmouk University, Irbid, Jordan

## Abstract

Breast cancer is the most diagnosed cancer among women around the world. The development of computer-aided diagnosis tools is essential to help pathologists to accurately interpret and discriminate between malignant and benign tumors. This paper proposes the development of an automated proliferative breast lesion diagnosis based on machine-learning algorithms. We used Tabu search to select the most significant features. The evaluation of the feature is based on the dependency degree of each attribute in the rough set. The categorization of reduced features was built using five machine-learning algorithms. The proposed models were applied to the BIDMC-MGH and Wisconsin Diagnostic Breast Cancer datasets. The performance measures of the used models were evaluated owing to five criteria. The top performing models were AdaBoost and logistic regression. Comparisons with others works prove the efficiency of the proposed method for superior diagnosis of breast cancer against the reviewed classification techniques.

## 1. Introduction

Breast cancer is a common cause of death and is the type of cancer that is widespread among women worldwide [1]. Many imaging techniques and tools have been developed for early detection and treatment of breast cancer and for reducing the number of deaths as a result of it [2]. There have been many breast cancer diagnosis methods that were used to increase diagnostic accuracy [3, 4].

In the last few decades, several data mining and machine-learning techniques have been developed for breast cancer detection and classification [5–7]. These approaches can be divided into three main stages: preprocessing, feature extraction, and classification.

In order to facilitate interpretation and benefit pursuit analysis, preprocessing of mammography helps to improve the visibility of peripheral areas and intensity distribution. For this reason, several methods have been reported [8, 9].

Feature extraction is an important step in breast cancer detection, where the main features help in discriminating benign tumors from the malignant ones. After this, several image properties, such as smoothness, coarseness, depth, and regularity, are extracted by means of segmentation [10]. Various transform-based texture analysis techniques are applied to convert the image into a new form using the spatial frequency properties of the pixel intensity variations. The common techniques are wavelet transform [11], fast Fourier transform (FFT) [12], Gabor transforms [13], and singular value decomposition (SVD) [14]. To reduce the dimensionality of the feature representation, principal component analysis (PCA) [15] can be applied.

Many works have attempted to automate the diagnosis of breast cancer based on machine-learning algorithms. Yap et al. [16] used three different methods of deep learning to detect the ultrasound of breast lesions: a patch-based LeNet, a U-Net, and a transfer learning approach with a pretrained FCN-AlexNet. Two different datasets are used. The first dataset consists of 306 images (60 malignant and 246 benign), and the second dataset consists of 163 images (53 malicious and 110 benign). The best *F*-measure results obtained by Yab were 91% and 89%, respectively. Qiao et al. [17] used the BI-RADS data system to improve diagnosis accuracy through ultrasound. In this work, the authors applied a genetic algorithm for the selection of features and the AdaBoost classifier to distinguish between benign and malignant tumors. Experiments were conducted on 138 tumors from the database using cross-checking of exit. The obtained accuracy was 93.48%. Wang et al. [18] applied the support vector machine (SVM) algorithm for breast cancer diagnosis in order to minimize the variability and increase diagnostic accuracy. In this work, receiver-operating characteristic curve ensemble (WAUCE) was used as a performance measure. The results of employing 12 different types of SVM were 97.89% for variation and 33.34% for accuracy. Amrane et al. [19] proposed two different classifiers (k-nearest neighbors (KNN) and Naïve Bayes (NB)) to diagnose breast cancer. The results showed that KNN achieved the highest accuracy of 97.51%, and the lowest accuracy of NB was 96.19%. Sun et al. [20] explored a multimodal deep neural network model by integrating multidimensional data (MDNNMD) to predict breast cancer. The obtained results show that there are still some issues in predicting the survival time of breast cancer with MDNNMD effectively. The proposed algorithm achieved an accuracy of 79.4%. The convolutional neural network improvement for breast cancer classification (CNNI-BCC) was applied to extract the relevant features from mammogram images [21]. The classification of the given features with CNNI-BCC achieved an accuracy of 90.50%.

Comparisons between neural networks and deep neural network algorithms with and without dimensional reduction techniques, and linear discrimination analysis were applied in [22] to classify the Wisconsin Diagnostic Breast Cancer (WDBC) dataset. The results showed that these algorithms performed well in terms of disease diagnosis and classification. The classification results proved the closeness of the accuracy of both previous models. In [23], the authors presented a comparison of three algorithms (Naïve Bayes, RBF network, and J48) on 683 breast cancer datasets with 10-fold cross-validation. The results showed that Naïve Bayes had the best accuracy of 97.36%, while RBF network and J48 achieved accuracies of 97.77% and 93.41%, respectively. Mondal et al. [24] explored the entropy method with four machine-learning algorithms to distinguish between normal tissues and breast cancer. The comparison of SVM, NB, KNN, and random forest (RF) indicated that SVM outperforms the others with an accuracy of 91.5%. Zhou et al. [25] exploited shear-wave elastography (SWE) data with a convolutional neural network (CNN) for breast cancer diagnosis using a database with 540 images, where 315 were malignant and 222 benign. The study achieved an accuracy of 95.8%, sensitivity of 96.2%, and specificity of 95.7%. In order to improve the accuracy of breast cancer diagnosis, Tamilvanan [26] applied the dimensionality reduction technique to five classifiers: multilayer perceptron, NB, radial basis function network, conjunctive rule, and KNN. Besides precision, recall, F-measure, accuracy, and ROC, new metrics were used, such as balanced classification rate (BCR) and Matthews correlation coefficient (MCC). The experiments revealed that the NB algorithm had the highest accuracy of 82%. Tapak et al. [27] compared and analyzed eight classifiers to predict breast cancer survival and metastasis. The classifiers used were AdaBoost, Naïve Bayes (NB), least-square SVM (LSSVM), random forest (RF), SVM, linear discriminant analysis, Adabag, and logistic regression (LR). The proposed models were applied to 550 patients. The deduced results demonstrate outperformance of SVM over the other machine-learning methods. In [28], the authors proposed the CRISP-DM methodology to analyze the WDBC dataset. The achieved best accuracy was for the SVM.

Tahmassebi et al. [29] applied magnetic resonance methods for detecting breast cancer in women (average age: 46.5 years, range: 25–70 years). Eight classifiers were used to categorize features, including linear SVM, linear discrimination analysis, logistic regression, decision tree, adaptive enhancement, and enhanced gradient extreme (XGBoost). To reduce the dimension, the authors applied various features selection techniques: survival-free redundancy (RFS) and survival of the disease (DSS). The rating accuracy was assessed with the area under the receiver operating characteristics curve (AUC). The best result was found with an AUC of 92%.

The Gabor wavelet has been proposed to extract features from mammography images in [30]. Several methods were used to classify radiographic images: C5.0 tree, SVM, artificial neural networks, Tree Quest, and CHAID. The comparison of the previous classifiers models demonstrates that SVM outperforms the other with an accuracy of 96%. Another deep learning algorithm was applied by Arau et al. [31] to recognize benign and malignant abnormalities. In this work, the authors used the convolutional neural networks (CNNs) to extract features. The given model was applied to a database with four classes (normal tissue, benign lesion, in situ carcinoma, and invasive carcinoma) and two classes (carcinoma and noncarcinoma). The sensitivity of this method was 95.6%.

In [32], the authors explored the tissue morphology in hematoxylin and eosin (H&E) stained breast cancer tissue microarray (TMA) samples using a machine-learning algorithm. The presented experiments showed that the morphological patterns in breast cancer can be explored using unsupervised machine learning.

Sharma et al. [7] applied three machine-learning algorithms to predict breast cancer. The experimental results gave an accuracy training ranging from 93% to 97%.

In order to ameliorate breast cancer histopathological image classification accuracy, Wei et al. [33] applied a deep CNN named BiCNN. The contribution of the authors was to propose a new technique to extract the features from mammography images. Radiya-Dixit et al. [34] developed a combined model with active feature extraction (CAFE) based on logistic regression (LR) techniques. In this work, the authors compared the proposed model to five machine-learning models. The best AUC was 91.8%.

In [35], the authors proposed an assistant tool based on deep learning to detect the breast cancer metastasis in lymph nodes. The developed tool demonstrates its effectiveness to identify micrometastases.  Table 1 summarizes selected literature on breast cancer diagnosis. It highlights the type of technique applied to select the features, the classifier model to distinguish between malignant and benign, the accuracy measure, and the database used to validate the proposed mode.

While the higher accuracy of the various methods applied for the breast cancer diagnosis, the use of Tabu search metaheuristic [43] to reduce the dimension features improves the performance of the applied algorithms. The present work compares five machine-learning techniques to distinguish between malignant and benign breast cancer. The techniques include KNN [44], Gaussian Naïve Bayes (GNB) [45], logistic regression (LR) [46], the extremely randomized tree (ET) [47], and adaptive boosting [48].

The remainder of this paper is organized as follows.  In Section 2, the material and methods are explained.  Section 3 summarizes the experimental results, and Section 4 presents the conclusions.

## 2. Materials and Methods

### 2.1. Datasets Used for Research

In this work, two databases were used to validate the proposed algorithms. In fact, those who work with models of medical field are aware that relying on just one data source can be problematic. In addition two different methods have been applied to extract the features for each dataset; in BIDMC-MGH dataset, the feature extraction is basically composed by nuclei segmentation and nuclei computation; however, in WDBC dataset, the features were extracted depending on nuclei computation. The WDBC is frequently used in the comparison of BIDMC-MGH.

The BIDMC-MGH dataset was created in collaboration with the two centers, MGH and BIMDC. In addition to this, the two partners proceeded based on standardized laboratory protocol and the same equipment in their study. The BIDMC-MGH dataset [5] is composed of 392 features based on shape, intensity, texture, and color. The dataset contains 116 breast biopsies from MGH and 51 breast biopsies from BIDMC diagnosed as DCIS or UDH. The first 116 samples are used for training and the other 51 samples for validation of the proposed models. The features of BIDMC-MGH were computed from the morphological and statistical features of the selection nuclear regions. Based on shape and measurement values, the morphological features were computed including perimeter, area, bounding rectangle fit ellipse, shape descriptors, and Feret's diameter. The statistical features computations depend on the intensity, the texture, and eight chosen colors. Using the statistical analysis, the mean, median, and standard deviation are computed for each feature per patient. All images are extracted from the following website: http://earlybreast.becklab.org/.

The features of WDBC are subtracted from digitized images of a fine needle aspirate of a breast mass (FNA), which describes features of the nucleus of the current image. The Wisconsin Diagnostic Breast Cancer (WDBC) database is composed of 569 observations, where 357 are benign and 212 are malignant breast masses. The 30 descriptive features produced from the three statistic values were computed based on ten FNA geometric measurements for each cell nucleus: radius, texture, perimeter, area, smoothness, compactness, concavity, concave point, symmetry, and fractal dimension of each mass.

### 2.2. Feature Selection

The feature selection is one of the most important steps in designing the classifier model. The main objective of feature selection operation is to get the best representation of the data which finds a lower dimensional representation of data. Usually high-dimensional representation of the data leads to degeneration in the performance of the used method. By considering that the redundant data should be removed and only the relevant feature will be used, the performance of the useful model can be improved or can be maintained and can simplify the complexity the applied model. In this context, the proposed feature selection method was presented. In this work, the Tabu search method based on rough set theory was introduced. The selection of the appropriate features depends on four stages: neighborhood search, diversification, shaking, and elite reduct. This process selects the informative information in order to discriminate between the normal and cancerous tissues; in addition, the large number of features increases the computing complexity. So the classification process will run slowly. Moreover, accurate diagnosis depends strongly on appropriate features being selected.

#### 2.2.1. Rough Set Theory Based Feature Reduction

Recently, rough set theory has proved its effectiveness to reduce the features of a given dataset [49]. Mainly, this theory is based on discernibility and attribute dependency [50] to evaluate the contribution of each attribute. In rough set theory, all the attributes are called the decision system which is composed of conditional attributes set *C* as input and decision attributes set *D* as output.

Assume *I*=(*U*, *A*) an information system, where *U* is a nonempty set of finite objects called universe of discourse and *A* is a nonempty set of attributes such that, for every *a* ∈ *A*, *a* : *U*⟶Va, where Va is the value set of *a*.

With any subset *P*⊆*A*, there is an associated equivalence relation called *P*-indiscernibility relation defined as follows: IND_*I*_(*P*)={(*x*, *y*) ∈ *U* × *U*/ ∀*a* ∈ *P*, *a*(*x*)=*a*(*y*)}. In other words, the objects *x* and *y* are indiscernible from each other by attributes from *P*. The equivalence classes of the *P*-indiscernibility relation are designated by [*x*]_*P*_.

Let *X*⊆*U*; the set of object *X* can be approximated based on *P*-lower and *P*-upper approximations denoted by $\underline{P}X$ and $\bar{P}X$, respectively, where $\underline{P}X=\left\{x/{\left[x\right]}_{P}|\subseteq |X\right\}$ and $\bar{P}X=\left\{x/{\left[x\right]}_{P}|\cap^{}|X|=|\emptyset \right\}$.

Assume *P* and *Q* are equivalence relations on *U*; the positive region *Q* with respect to *P* is defined as ${\text{POS}}_{P}\left(Q\right)=\cup_{X\in U/Q}\underline{P}X$. Based on positive region, the degree of dependency of *Q* frm *P* is defined as

(1) $$\begin{array}{c} \gamma_{P}\left(Q\right)=\frac{\left|{\text{POS}}_{P}\left(Q\right)\right|}{\left|U\right|}, \end{array}$$

where |*U*| defines the cardinality of the set *U*.

The computing of the attribute reduction for a large dataset is very expensive due to the existence of a set of reduction attributions. As a result, an alternative tool is necessary to overcome this concern.

#### 2.2.2. Tabu Search Based on Feature Reduction

Tabu search (TS) [43] is a heuristic method of local search used to solve complex and many large optimization problems. It is classified as a local search method with adaptive memory.

In fact, its main feature is to memorize solutions or information search processes visited during the search to explore the research space beyond local optimality. An adaptive memory algorithm can effectively generate a neighborhood solution from the current solution and accepts the best solution even if it does not improve the current solution. The memory contains a list of recently visited solutions and avoids cycling and falling back into permanence in the local optimum. This simple process then allows escape from the local optimum so other areas of the solution space can be visited. To summarize, we can say that, at each iteration, the process continues to explore the solution space even though it does not improve the solution.

The TS-based attribute reduction [50] is mainly designed using the four strategies.

**Figure 1:**
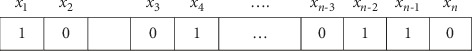
Sample of trial solution.

**Figure 2:**
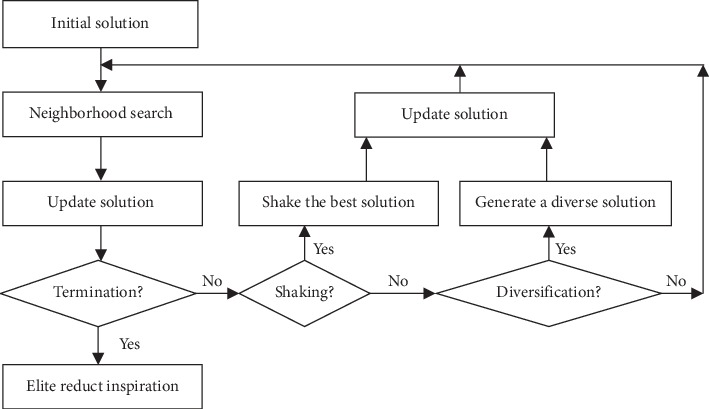
Feature selection strategy.

Strategy 1: neighborhood search
    The basic goal of this strategy is to explore the solution around the current solution, besides it avoids generating a solution recently used in the Tabu List (TL).
    Assume *yi* is the trial solution of the neighbors of *x*, where *I* ∈ [1, *m*], and *m* is the number of trial solutions. An example of binary representation of a trial solution *x* is shown in Figure 1. The component of each vector equals zero or one according to the presence of each attribute in trial vector.
    The extreme trial solutions are the vectors that contain zero or one for all the positions. The updating of the trial solutions is based on applying mutation multipoints, where the number of positions is generated randomly.
Strategy 2: diversification
    In order to explore the space solution widely, the diversification will be applied. The diverse solution can be composed of the attributes not invoked during the generation of trial solutions. The attributes with a low appearance will be selected with a probability inversely proportional to that appearing in the generation of the trial solutions.
Strategy 3: shaking
    The purpose of this strategy is to optimize the current best solution. This procedure begins by deleting the contained attributes of the best solution one by one without any reducing of the dependency degree of *γ*_ (*x*best).
    In other words, the updating of *x*best is approved only if the value of its dependency degree increased or kept the same value after removing an attribute that composes the solution.
Strategy 4: elite reduct
    This strategy aims to find the minimal set attributes. Basically, this process depends on the previous strategy. In other words, the set of attributes involved in the minimal reduced vector is composed of the intersection of the list of vectors formed by optimizing the best solution.  Figure 2 depicts the Tabu search based feature selection.

### 2.3. Machine-Learning Techniques

Often, the selection of classifier is one of the challenges in fielding of machine learning for the real applications. The choice of the classifiers algorithms depends on many factors. Some parameters are the complexity, the accuracy, the types of features/labels, and the suitability for certain sizes and dimensions of datasets.

The benefits of applying the Naïve Bayesian classifier are that it is less sensitive to the outliers, performs with less parameter, and also can outperform more alternative classifiers for small sample sizes.

The KNN is a supervised classifier that works well with small-size dataset. It is robust to a noisy data and requires one hyperparameter. Moreover, this technique can be used for both classification and regression problems.

Logistic performs better on a dataset with a small size. The effectiveness of the technique is to require few computational resources and does not need any tuning.The major strength of AdaBoost technique is its insusceptibility to overfitting problem.

The Extremely Randomized Trees (ET) model is relative to data with small number of samples and works with a reduced computational complexity.

#### 2.3.1. K-Nearest Neighbors Algorithm

KNN was proposed by Cover and Hart and is considered one of the most successful machine-learning models to solve both classification and regression problems. The KNN technique is based on feature similarity measures. The aim of this method is to assign weights to the contributions of neighbors by assigning nearer neighbors with more weight than the more distant ones. The weight can be found based on the distance between instances. In general, distance measures can be standard Euclidean distance, Hamming distance, Manhattan distance, and Minkowski distance.

#### 2.3.2. Gaussian Naïve Bayes

The Gaussian Naïve Bayes (GNB) classifier is a probabilistic machine-learning model and one of the most successful algorithms for classification tasks in medical image analysis [51]. The key insight of the classifier is conditional probability to classify data. The GNB algorithm is an example of NB algorithm where the features have continuous values and follow a Gaussian distribution.

#### 2.3.3. Logistic Regression

LR, which is a statistics-based machine-learning algorithm, is generally applied for binary classification problems (problems with two class values). It is an effective method of modeling a categorical outcome (binomial/multinomial) with one or more independent variables. Unlike linear regression, which is used to study relationships between two continuous (quantitative) variables, LR is used to ascertain a probability value that can be mapped to two or more discrete classes.

#### 2.3.4. Extremely Randomized Trees Classifier (ET)

The ET is similar to RF, based on extreme randomization of the tree construction algorithm and disabling pruning operator. Ultimately, the main differences to RF are that it makes a small number of randomly chosen split points and it uses the whole learning sample. Besides better generalization abilities, the main strength of the ET technique is the reduction of the computational complexity.

#### 2.3.5. AdaBoost Classifier (AB)

Recently, the adaptive boosting gets a great interest in machine-learning competitions. It is proposed by Freund and Schapire in 1996. The technique focuses on building a classifier composed of a number of weak classifiers using the following equation:

(2) $$\begin{array}{c} F\left(x\right)=\text{sign}\left(\sum_{i=1}^{n}w_{i}f_{i}\left(x\right)\right), \end{array}$$

where *w*_*i*_ is the weight of the weak classifiers *f*_*i*_.

This technique is based on the low correlation between the classifiers which significantly improve the accuracy of weak learning algorithms. At each step, the weights of the training examples were computed for each used model. The classifiers with low performances were kept and combined to produce the final results.

### 2.4. Evaluation Parameters

Various metrics are used to evaluate machine-learning algorithms. In this study, the useful metrics are accuracy, sensitivity, precision, *F*-score, and AUC.

Accuracy is the measure of correct prediction of the classifier and provides general information about how many samples are misclassified. It is defined as

(3) $$\begin{array}{c} \text{accuracy}\left(\%\right)=\frac{\text{TP}+\text{TN}}{\text{TN}+\text{FP}+\text{TP}+\text{FN}}\times 100, \end{array}$$

where TP, FP, TN, and FN are the numbers of true positives, false positives, true negatives, and false negatives, respectively, when the classifier is predicted.

Sensitivity is the ratio of the number of correctly predicted benign tumors to the total number of benign tumors:

(4) $$\begin{array}{c} \text{sensitivity}\left(\%\right)=\text{TPR}=\frac{\text{TP}}{\text{TP}+\text{FN}}\times 100. \end{array}$$

Specificity is the proportion of actual malignant tumors that are classified as malignant by the model:

(5) $$\begin{array}{c} \text{precision}\left(\%\right)=\frac{\text{TP}}{\text{TP}+\text{FP}}\times 100,\\ F1\text{score}\left(\%\right)=\frac{\text{precision}\times \text{sensitivity}}{\text{precision}+\text{sensitivity}}\times 100. \end{array}$$

In this study, besides the above metrics, the receiver-operating characteristic (ROC) graph AUC was employed.

## 3. Results and Discussion

To provide more evaluations, the proposed classifiers for breast cancer diagnosis were analyzed to study the effect of the TS technique on accuracy and to also compare it to techniques used in other works.

### 3.1. BIDMC-MGH Dataset

In this study, we compare the performance of five machine-learning techniques for the BIDMC-MGH database with 392 features and the WDBC database with 32 features. Several metrics were used to quantitatively evaluate the diagnostic performance of the classifiers. For the metrics described above, a higher percentage indicates a better classification accuracy. Note that the AUC is a powerful metric for the classification of performance.

From the BIDMC-MGH dataset, 116 samples from the MGH hospital are used for training and 51 samples from the BIDMC hospital are used for testing. The TS was used to select features from all 392 features.  Table 2 presents the comparison of the five classifiers using all features and without applying the TS feature selection. The best result to discriminate between malignant and benign cancer was related to using linear regression. As can be seen, all the applied classifiers for the BIDMC-MGH dataset did not exceed 83% in classification performance.

**Figure 3:**
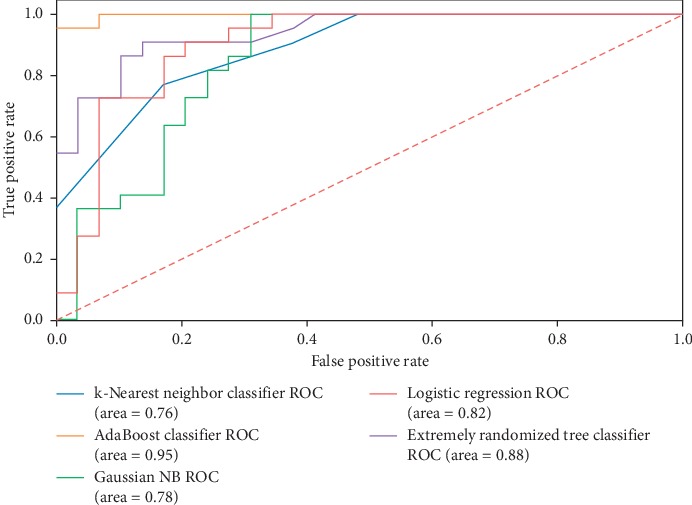
Analyzing the obtained results via different classifiers on BIDMC-MGH.

Table 3 lists the five metrics used for all classification techniques. As can be seen from Table 3, the AdaBoost presents the best models regarding accuracy, with an AUC score equal to 95%. As is known, AUC values provide a more accurate scoring measure than the other metrics based on true/false ratio.  Figure 3 illustrates the comparison of five machine-learning algorithms for all the applied metrics.

**Figure 4:**
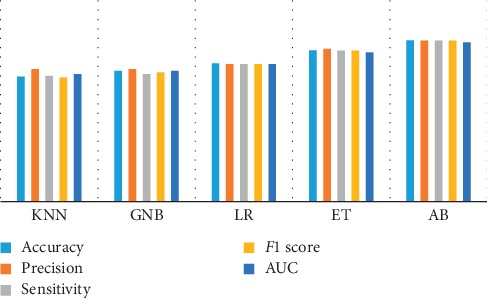
The ROC curve of the applied machine-learning algorithms.

**Figure 5:**
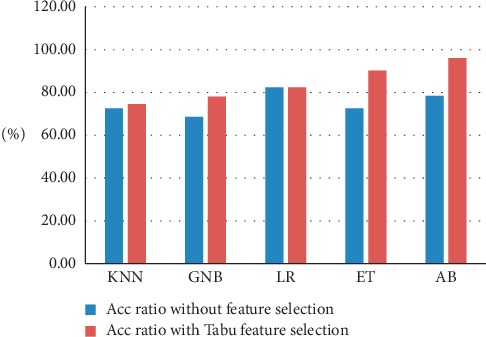
The effect of feature selection on accuracy on BIDMC-MGH.

Table 4 shows the comparison of the proposed machine-learning classifiers to other models using the same database. Performance evaluation is given by AUC ratio. The derived results show performance superiority of the AdaBoost classifier when compared to the models proposed in [5, 34].  Figure 4 shows the receiver-operating characteristic curves of the applied machine-learning algorithms in this experiment. The findings prove that Tabu feature selection can improve the accuracy of the applied classifiers. The feature selection process has an impact on most of the applied classifiers method.  Figure 5 shows the effect of Tabu feature selection on the classifier accuracy. The TS technique reduces the number of features from 392 to 25. As a result, the obtained data become more understandable and easier to study.

### 3.2. WDBC Dataset

The proposed method was applied to the WDBC dataset. The effect of Tabu feature selection on accuracy is again analyzed in this section. The derived result will be compared to a list of machine-learning classifiers included in the review of the first section. The performance evaluation is measured by the accuracy metric for the comparison analysis because the methods presented in Table 1 used this evaluation criterion.

In this experiment, the proposed classifiers were first applied to the WDBC dataset with all 32 features and then applied to the obtained TS-based features.

Table 5 illustrates the performance of the used classifiers before applying the TS method for feature selection, whereas Table 6 presents the evaluations performance of the machine-learning classifiers with Tabu features.

**Figure 6:**
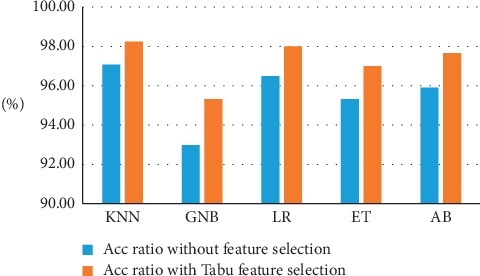
Effect of feature selection on accuracy classifiers on WDBC.

Figure 6 presents the effect of feature selection on the accuracy evaluation of the used classifiers.  Table 7 shows the comparison of the proposed method with other methods in the literature. When we kept accuracy as the only criterion for evaluating performance, KNN was the best classifier model for classification of WDBC, whereas the linear regression method was the best classifier using the AUC metric.

## 4. Conclusions

Throughout this work, we developed an automated machine-learning technique for breast cancer diagnosis. The proposed method was based on TS for the feature selection process, and five machine-learning algorithms were implemented to discriminate between malignant and benign cancer. The metrics for diagnosis of the BIDMC-MGH and WDBC datasets were evaluated using five evaluation criteria. Although the accuracy was used to evaluate the performance of the implemented models for breast cancer diagnosis, the AUC value along with the sensitivity, precision, and *F*1-score can in turn examine the evaluation process. In many cases, the derived conclusions based on accuracy metrics were totally different to those when using the AUC measure. We have shown via WDBC experiment comparisons that, in terms of AUC, KNN is the best classifier. However, this conclusion is not correct when considering accuracy. Moreover, the proposed classifiers based on the TS method demonstrate performance superiority over the other models. Future work includes extending the proposed algorithm for breast cancer to find the grade of malignant diagnosis and to apply the statistical methods to others pathology fields.

## Figures and Tables

**Table 1:** Summary of machine-learning algorithms for breast cancer diagnosis.

Author	Feature	Algorithm	Accuracy (%)	Dataset
Asri et al. [36]	FNA	SVM	97.13	UCI
Ivančáková et al. [28]	FNA	SVM	97.66	WDBC
Mondal et al. [24]	Entropy	SVM	91.5	Gene Expression Omnibus
Ghasemzadeh et al. [30]	Gabor wavelet	SVM	96	Mammography (DDSM)
Ayoub Shaikh and Ali [37]	Wrapper subset eval	SVM	97	Breast Cancer Digital Repository (BCDR)
Wang et al. [18]	Full features	SVM	33.34	SEER
Mengjie Yu [38]	Concave points	SVM	99.77	UCI
Wei et al. [33]	BiCNN	CNN	97	BreaKHis
Bejnordi et al. [39]	Morphology	CNN	92	WSIs
Arau et al. [31]	Full features	CNN	83	Histology Dataset
Yap et al. [16]	FCN-alexnet	CNN	98	B&K Medical Panther 2002 and B&K Medical Hawk 2102 US systems
			92	
Ting et al. [21]	Wise	CNN	90.50	Digital Mammogram
Zhou et al. [25]	CNN	CNN	95.8	SWE data
Sun et al. [20]	mRMR	Deep neural network	18.7	METABRIC/MDNNMD
Kaur et al. [40]	CNN	MLP	86	Mini-MIAS
Joshi et al. [22]	Scaling	NN	96.47	WDBC
Radiya-Dixit et al. [34]	Computational method	LR	91.8	BIDMC-MGH
Tahmassebi et al. [29]	Volume distribution	LR	92	WDBC
Braman et al. [41]	Heterogeneity	LR	93	ISPY1-TRIAL
Maysanjaya et al. [42]	Wrapper	NB	99.27.	UCI
Chaurasia et al. [23]	FNA	NB	97.36	WDBC
Tamilvanan [26]	Dimensionality reduction	NB	82	WDBC
Qiao et al. [17]	BI-RADS	AdaBoost	93.48	138 pathologically proven breast tumors
Turkki et al. [32]	Morphological	KNN	95	FinProg
Amrane et al. [19]	FNA	KNN	97.51	WDBC

**Table 2:** Classifier results on BIDMC-MGH without Tabu feature selection.

Model	Accuracy (%)	Precision (%)	Sensitivity (%)	*F*1 score (%)	AUC (%)
KNN	72.54	75	73	73	74
GNB	68.62	70	69	69	69
LR	82.35	83	82	83	83
ET	72.54	72	73	72	69
AB	78.43	79	78	79	77

**Table 3:** Classifier results on BIDMC-MGH with Tabu feature selection.

Model	Accuracy (%)	Precision (%)	Sensitivity (%)	*F*1 score (%)	AUC (%)
KNN	74.50	79	75	74	76
GNB	78	79	76	77	78
LR	82.35	82	82	82	82
ET	90.19	91	90	90	89
AB	96.07	96	96	96	95

**Table 4:** Comparison of proposed method and other methods on BIDMC-MGH.

Model	AUC (%)
L1-regularized LR [5]	85.8
L1-regularized LR with active feature [5]	89.7
LR with early stopping and active features [34]	88.4
CAFE model [34]	91.8
*Proposed model*	**95**

**Table 5:** Classifier results on WDBC without Tabu feature selection.

Model	Accuracy (%)	Precision (%)	Sensitivity (%)	*F*1 score (%)	AUC (%)
KNN	97.07	97	97	97	97
GNB	92.98	93	93	93	93
LR	96.49	97	96	97	97
ET	95.32	96	95	95	96
AB	95.90	96	96	96	96

**Table 6:** Classifier results on WDBC with Tabu feature selection.

Model	Accuracy (%)	Precision (%)	Sensitivity (%)	*F*1 score (%)	AUC (%)
KNN	98.24	98	98	98	97
GNB	95.32	95	95	95	95
LR	98	99	99	99	98
ET	97	97	97	97	97
AB	97.66	98	98	98	97

**Table 7:** Comparison of proposed method and other methods on WDBC.

Model	Accuracy (%)
NN [22]	96.47
KNN [19]	97.51
LR [29]	92
NB [23]	97.36
NB [26]	82
*The proposed model*	**98.24**
