# Corrigendum to “Tabu Search and Machine-Learning Classification of Benign and Malignant Proliferative Breast Lesions”

**DOI:** 10.1155/2020/7638969

**Published:** 2020-06-30

**Authors:** Habib Dhahri, Ines Rahmany, Awais Mahmood, Eslam Al Maghayreh, Wail Elkilani

**Affiliations:** ^1^College of Applied Computer Sciences (CACS), Al-Muzahimiyah Branch, King Saud University, Riyadh, Saudi Arabia; ^2^Faculty of Sciences and Technology of Sidi Bouzid, University of Kairouan, Kairouan, Tunisia; ^3^Computer Science Department, Yarmouk University, Irbid, Jordan

In the article titled “Tabu Search and Machine-Learning Classification of Benign and Malignant Proliferative Breast Lesions” [1], author Dr. Eslam Al Maghayreh was not affiliated to “College of Applied Computer Sciences (CACS), Al-Muzahimiyah Branch, King Saud University, Riyadh, Saudi Arabia” which is incorrect. The corrected list of affiliations is shown in the author information above.
